# Internal Jugular Vein Duplication: A series of Seven Cases and Review of Literature

**DOI:** 10.22038/ijorl.2024.82759.3789

**Published:** 2025

**Authors:** Indu Shukla, Ashish Agarwal, Rimsha Changanath Kader

**Affiliations:** 1 *Department of ENT, ABVIMS & Dr RML Hospital New Delhi-110001India.*

**Keywords:** Neck Dissection, Duplication, Jugular vein

## Abstract

**Introduction:**

Internal Jugular Vein (IJV) is an important landmark for Head and Neck surgeons during oncological clearance of disease from neck and microvascular reconstruction as well as for the intensivist during central line insertion. Detailed knowledge of the IJV anatomy and its variations is important to avert any catastrophic complications during surgery.

**Materials and Methods:**

Data of 350 patients was recorded prospectively and analysed over a period of two years and presence of IJV duplication was documented as percentages**.**

**Results:**

A total of 350 patients with diagnosed oral cavity carcinoma were included who underwent neck dissection out of which seven patients were identified with Internal Jugular Vein duplication making it an institutional clinical prevalence of around 2%.

**Conclusion:**

IJV duplication is inadvertently found intra operatively on maximum number of occasions therefore to avoid the risk of iatrogenic injury and undesired complications, preoperative imaging should be carefully assessed while planning the patient for surgery.

## Introduction

IJV is the principal deep vein that drains the majority of head and neck region.

It begins below the jugular foramen as continuation of sigmoid sinus and ends posterior to medial part of clavicle by joining subclavian vein to form the brachiocephalic vein (1,2).

Neck dissection is the cornerstone of management of head and neck cancer for loco regional clearance of disease (3,4).

IJV represents a relevant surgical anatomical landmark for adjacent structures such as carotid artery, vagus nerve, spinal accessory nerve (SAN) and jugular chain of lymph nodes.

The identification and preservation of IJV and SAN is important in reducing surgical complications and post operative morbidity. 

Complex embryological development of vascular system often results in clinically relevant anomalies (5,6).

IJV can present with certain anomalies like duplication and fenestration. In the former, vein bifurcates into two segments separately draining into subclavian vein forming a reversed pattern whereas in the latter, IJV bifurcation reunites proximal to subclavian vein (7-9). Hence identification of these anatomical variations are useful to avoid unexpected surgical complications during neck dissections and central venous catheterization.

Anatomical anomalies of IJV are seldom reported. Prevalence of IJV duplication is generally limited to few case reports. Till date 24 cases of IJV duplication have been reported in the literature (5,9). Keeping in mind the scarcity of literature, we report a case series of 7 patients with IJV duplication found incidentally during neck dissection.

## Materials and Methods

Patients (both males and females) with diagnosed oral squamous cell carcinoma undergoing surgical resection of primary lesion with neck dissection were included in the study. Data of 350 patients was recorded over a period of two years in the form of percentages.

## Results

A total of 350 patients presenting to the ENT OPD with confirmed diagnosis of oral cavity squamous cell carcinoma were subjected to Wide local excision of lesion with neck dissection aiming at loco regional clearance of disease. Incidentally, in seven out of 350 cases, during neck dissection, Internal jugular vein duplication was found. 

**Fig 1:**
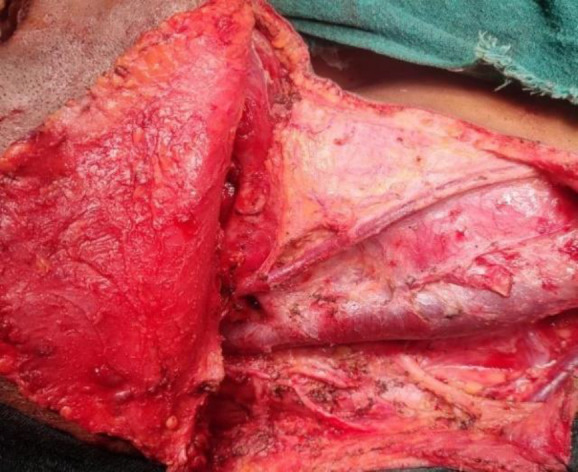
Duplication of IJV on right side

**Fig 2:**
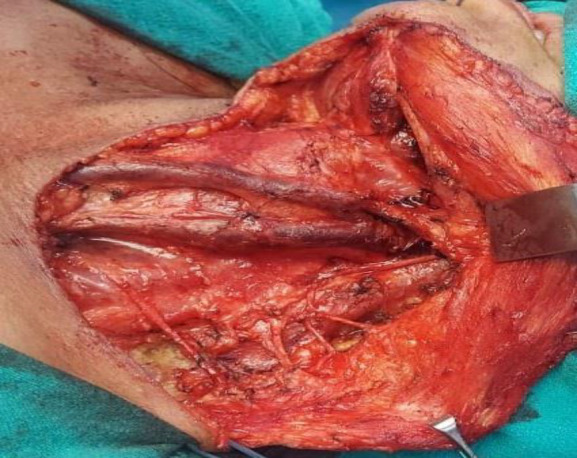
Duplication of IJV on left side

It was noticed on the right side in 4 cases and on the left side in 3 cases. Figure 1 and 2 illustrate duplicated internal jugular vein on right side and left side respectively.

In all the cases of IJV duplication, the duplication started beneath the intermediate tendon of digastric muscle and both the segments then continued independently to drain behind the clavicle. (Figure 1 and 2).

**Fig 3:**
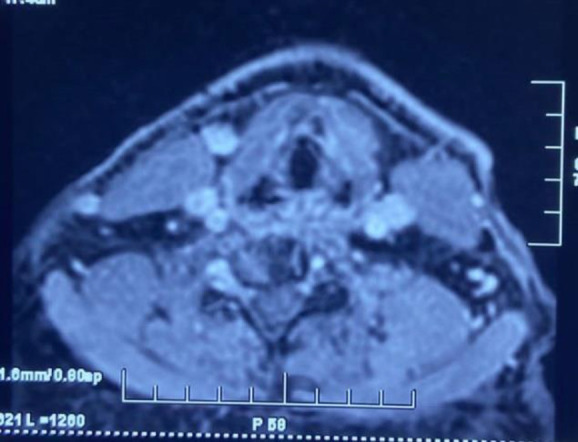
CECT Neck showing Duplicated IJV on Right side

In all the cases a preoperative CECT – Face and Neck with puffed cheek was performed. But the presence of IJV duplication was not reported in any of the seven cases. On tracing the films thoroughly in the postoperative period, a duplicated IJV could be seen as illustrated in Figure 3. On calculating, the prevalence of IJV duplication as an anatomical variation atour institute, it was found to be around 2 %.

## Discussion

IJV is a major vein of head and neck collecting blood from cranium, face and neck (2).

Embryologically IJV develops from ventral pharyngeal vein which drains into precardinal vein which contributes to lower part of IJV formation as the neck elongates. Various hypothesis have been postulated for IJV duplication mainly venous hypothesis where persistence of two venous channels during condensation of embryonic venous plexus could lead to duplication (10), whereas neuronal hypothesis suggest that branching of IJV is due to entrapment of accessory nerve within the developing embryonic venous plexuses (consistent with cases where SAN bisects IJV) (10). 

In bony hypothesis duplication of IJV is related to duplication of jugular foramen during ossification. Finally muscular hypothesis suggest duplication of IJV due to posterior belly of omohyoid muscle (2,7,9,11). Prevalence is merely based on case reports and small case series often limited to 1-2 cases. Prades JM et al estimated clinical prevalence of IJV duplication to be 0.4 % and Wang et al estimated prevalence of IJV duplication as 0.9% (9,10). In our case series, total of 7 cases of IJV duplication were found in over 350 neck dissections performed over a period of two years. Hence our institutional prevalence was 2 % which is considerably more than the reported prevalence so far. The strategic location and the consistent anatomical course of IJV makes it an important anatomical landmark during head and neck surgery involving neck dissection, reconstruction with free flaps and central venous catheterization. The close association of SAN with IJV anteriorly and posteriorly in 56-90% and 10-44% of cases respectively has been seen, putting the nerve at risk of injury especially where duplication of IJV is present (12). During IJV duplication, posterior segment lies posterior to SCM, putting it at risk of injury while clearing level IV during neck dissection. The relatively superficial position of the anterior segment puts the vessel at risk of injury during initial stages of neck dissection. Preservation of anterior segment is imperative (especially in absence of an EJV) as it receives all the tributaries that can be used for end-to-end anastomosis for the free flap (4).

Sanchez et al suggested anatomical variation may cause inadvertent injury of IJV leading to intra operative blood loss, obscuring the operative field and making clearance of lymph nodes difficult; therefore, it is important that the operating surgeon should be aware of IJV anatomical variation (5,13).

IJV duplication can be diagnosed preoperatively by CECT or magnetic resonance imaging of neck performed as part of preoperative staging of patients. These scans are also helpful in academic institutions where the residents can be taught to appreciate this anatomical variation and be mindful of it during neck dissection (4). In our case series, we could make preoperative diagnosis of IJV duplication in 2 patients based on imaging, rest of the cases were evinced intraoperatively by visualization making it the most common method of diagnosis reported in literature till date (14).

Right IJV is usually larger than left IJV since it drains blood from superior sagittal sinus. Mumtaz and Singh reported that more than two third of variation of IJV occurs on left side whereas in our case majority of variations were seen on the right side (5,14). Few symptoms like neck swelling, dysphagia have been reported in patients with IJV duplication but all patients included in our case series were found to be asymptomatic (9,15-18). 

Our study highlights the importance of IJV anatomical variation and surgical risks associated with it. Though it is a relatively rare clinical entity, the operating surgeon should be mindful of such variation while performing neck dissection to prevent inadvertent injury and unanticipated blood loss. 

## Conclusion

 Internal Jugular vein is the principal vein of neck. Anatomical variations of IJV especially IJV duplication is rare and needs to be kept in mind to minimize risk of injury during neck surgeries, Central venous line insertion and microvascular anastomosis during free flap reconstruction.

## Abbreviations

IJV- Internal Jugular Vein
